# Experiences of hospital-based multidisciplinary team meetings in oncology: An interview study among participating general practitioners

**DOI:** 10.1080/13814788.2017.1323081

**Published:** 2017-05-30

**Authors:** Peter Pype, Fien Mertens, Jeanluc Belche, Christiane Duchesnes, Laurence Kohn, Marij Sercu, Myriam Deveugele

**Affiliations:** ^a^ Department of Family Medicine and Primary Healthcare, Ghent UniversityGhentBelgium; ^b^ Département Universitaire de Médecine Générale, Faculté de Médecine, Université de LiègeLiegeBelgium; ^c^ Belgian Health Care Knowledge Centre (KCE)BrusselsBelgium

**Keywords:** Interdisciplinary communication, patient care team, general practitioners, medical oncology

## Abstract

**Background:** Cancer care has become complex, requiring healthcare professionals to collaborate to provide high-quality care. Multidisciplinary oncological team (MDT) meetings in the hospital have been implemented to coordinate individual cancer patients’ care. General practitioners (GPs) are invited to join, but their participation is minimal.

**Objectives:** Aim of this study is to explore participating GPs’ perceptions of their current role and to understand their preferences towards effective role execution during MDT meetings.

**Methods:** In May to June 2014, semi-structured interviews (*n* = 16) were conducted involving GPs with MDT experience in Belgium. The analysis was done according to qualitative content analysis principles.

**Results:** Attendance of an MDT meeting is perceived as part of the GP’s work, especially for complex patient care situations. Interprofessional collaborative relationships and the GP’s perceived benefit to the MDT meeting discussions are important motivators to participate. Enhanced continuity of information flow and optimized organizational time management were practical aspects triggering the GP’s intention to participate. GPs valued the communication with the patient before and after the meeting as an integral part of the MDT dynamics.

**Conclusion:** GPs perceive attendance of the MDT meeting as an integral part of their job. Suggestions are made to enhance the efficiency of the meetings.

KEY MESSAGES

- MDT meetings coordinate the efforts of healthcare providers in cancer patient care.
- General practitioners are willing to make efforts to participate in MDT meetings in case of complex patient situations.
- Continuity of information flow and good interprofessional relationships are facilitators for general practitioners’ participation in MDT meetings.

## Introduction

Evolutions in oncology have made cancer care more complex, resulting in the need for interprofessional collaboration. Internationally multidisciplinary team (MDT) meetings in oncology have been implemented in hospitals to allow specialists from different disciplines addressing the corresponding care coordination and communication challenge, including the general practitioner (GP), to form the best possible team to achieve optimal patient care [1,2]. The purpose of these MDT meetings is to develop a strategic plan for diagnosis, treatment and follow-up, and to discuss the overall care of an individual patient. The European Partnership Action against Cancer (EPAAC) described MDT meetings as:

Multidisciplinary team meetings [which] are an alliance of all medical and healthcare professionals related to a specific tumour disease whose approach to cancer care is guided by their willingness to agree on evidence-based clinical decisions and to coordinate the delivery of care at all stages of the process, encouraging patients in turn to take an active role in their care [1].

The use of MDT meetings often leads to better care [3,4]. GPs’ involvement in the meetings leads to better communication between hospital-based specialists and GPs, enhancing continuous care [5].

In Belgium, MDT meetings have been organized since 2003 to facilitate the development with specialists from different disciplines a strategic plan of diagnosis, treatment and follow-up and to discuss the overall care of an individual patient. These meetings are hospital based and attended by several medical specialists, e.g. oncologists, radiotherapists, radiologists, pathologists, general surgeons, urologists, gynaecologists, haematologists, pneumologists, gastroenterologists [6]. They have been identified as an essential step in the clinical pathway of each new cancer patient and whenever the treatment plan diverges from guidelines. The GP’s participation is officially established, although there is no real task description. Participation is rewarded by financial incentives. Nevertheless, the GP participation is currently minimal (from 0 to 4% in 2014) [6].

The literature describes general barriers for participation for the different disciplines involved, including workload [7], attendance problems [8,9], logistics and organizational aspects [7,5,10], communication and information problems [5,11]. The specific and personal views of GPs regarding participation and role execution during these multidisciplinary meetings are, however, unclear.

This research aims to explore the GPs’ motivation towards participating in MDT meetings by answering the following questions:

1. How do GPs perceive their current role and experiences regarding MDT meetings?
2. What are their preferences and expectations towards effective role execution regarding MDT meetings?

## Method

A qualitative research methodology was chosen as it aims to understand the lived experiences of people. Semi-structured interviews are an appropriate method to elicit participants’ personal experiences and preferences. Interviews may be preferred over focus group discussions when personal or sensitive issues are at stake.

### Sample

We purposefully selected GPs who had participated in at least one MDT meeting in the last five years to learn about their experiences. According to the qualitative approach, we built a sample with maximum diversity regarding language (Dutch and French-speaking GPs); age; gender and practice organization; regions and density of hospitals organizing MDT meetings; and hospital size.

Within the areas selected, representatives of the local GP circles and local GPs’ peer review groups were contacted and asked if they had attended a minimum of one MDT meeting during the last five years and if they were willing to be interviewed. Snowball sampling of other GPs was done. For each of the areas two GPs were selected leading to an initial sample of 16 GPs.

Additional sampling was scheduled after the analysis in case data saturation was not reached.

### Data collection tool

The Belgian Health Care Knowledge Centre delivered the literature-based interview guide and the Dutch and French research teams adapted the interview guide after discussion [6].

The main themes comprised GPs’ experience with and their perceived role and preferences towards the MDT meeting. Each research team performed a pilot test of the guide in their native language by interviewing one participant. After this, the interview guide was adapted and finalized for use (Appendix A).

### Data collection process

FM, JB and CD conducted the interviews in May and June 2014 at each interviewee’s practice. Informed consent was obtained. All interviews were audio-recorded and transcribed verbatim.

### Analysis

Content analysis of the transcripts was done using NVivo 10 software. In the first phase, a coding scheme was built, based on the themes of the interview guide and discussion of the first interviews’ analysis by both research teams. Subsequently, every interview was coded by two researchers independently and discussed in pairs. Refining of the codebook was done during further content analysis and regular discussions between both research teams. Discussions were held between researchers of both teams to crosscheck the coding results. In the second phase, the main findings and the meaning of the results were discussed during meetings between the two teams.

Analysis of the final interviews revealed no new themes. Therefore, we concluded that we had reached data saturation and an additional sampling procedure was not necessary.

Ethical approval was obtained from the Comité d’Ethique Hospitalo-Facultaire Universitaire of Liège and the central ethical committee of the University Hospital Ghent (no. B670201421076).

## Results

### Sample description

Sixteen GPs were interviewed, of whom six were female. Half of the participants were Dutch speaking; half were French speaking. The mean age was 48.75 years (range: 29–67 years). Five GPs were working solo and four in pairs. The remaining seven GPs worked in group practices, of which three were in a mono-disciplinary and four in a multi-disciplinary practice. Three of the latter multi-disciplinary practices had a capitated payment system. Sample characteristics are shown in Table 1.

**Table 1.** Sample characteristics.

Participant	Language	M/F	Age	Practice	Payment model: capitation/fee-for-service (FFS)
GP 1	Dutch	F	55	Group: mono-disciplinary	FFS
GP 2	Dutch	M	42	Group: multi-disciplinary	FFS
GP 3	Dutch	F	29	Duo	FFS
GP 4	Dutch	M	38	Solo	FFS
GP 5	Dutch	F	61	Duo	FFS
GP 6	Dutch	M	58	Solo	FFS
GP 7	Dutch	M	63	Solo	FFS
GP 8	Dutch	M	47	Group: mono-disciplinary	FFS
GP 9	French	F	56	Duo	FFS
GP 10	French	F	38	Solo	FFS
GP 11	French	M	37	Group: multi-disciplinary	Capitation
GP 12	French	M	39	Solo	FFS
GP 13	French	M	67	Group: multi-disciplinary	Capitation
GP 14	French	F	48	Duo	FFS
GP 15	French	M	62	Group: mono-disciplinary	FFS
GP 16	French	M	40	Group: multi-disciplinary	Capitation

### Four core themes

After analysis, four core themes emerged: (1) GPs’ perceived role and input in MDT meetings; (2) GPs’ perceived interactions with other MDT meeting participants; (3) GPs’ role as communicators and executors of meeting decisions regarding the patient; and (4) organizational issues for the participation of the GP in MDT meetings.

*(1) GPs’ perceived role and input in MDT meetings*. GPs in our study had a clear opinion about their role in the meeting, based upon their specific position and role in healthcare: they share a history of care with the patient, resulting in a repertoire of knowledge managed by the GP. This repertoire contains medical facts (e.g. previous diseases), social facts (e.g. the home care situation) and personal facts (e.g. a patient’s way of coping with misfortune) that can be brought to the MDT meeting to clarify the patient’s situation.

Some participants, however, hesitated to bring this latter information into the meeting that was sometimes perceived as a medical specialist meeting.

Some GPs positioned themselves as the patient’s representative because of their access to this kind of information.

Others felt they were appointed as the representative by the patient’s explicit demand that they participate in the MDT meeting. According to these GPs, patients considered the GP’s participation as a reassurance of good care. In these cases, the intensely personal relationship between the GP and the patient resulted in the sense of moral responsibility for the GP to address a specific patient request.

For most participants, complex medical situations (e.g. multimorbidity) and complex home care situations (e.g. absence of family support) especially prompted the GP to attend the MDT meeting and to participate in the deliberation process. The main reason was the direct impact of the MDT conclusions on the GP’s work, such as organizing home care (in the case of home care complexity) or acquiring new medical knowledge (in the case of medical complexity). Regarding straightforward cases, other ways of information sharing were sought, like calling the medical specialist. Only a minority of GPs declared that they tried to attend even the meetings on straightforward cases.

In early disease stages, when discussions are mostly about curative treatments, GPs feel less competent in playing a significant role. During the latter, advanced palliative stages, they feel they have a more important role.

(2) *GPs’ perceived interactions with other MDT meeting participants*. Participating GPs felt welcome at the MDT meeting. Most of the time, they experienced their participation as appreciated and their contribution was respected. The reciprocal appreciation created the feeling of being part of a team, facilitating active GP involvement. This was reported more in relation to smaller hospitals with well-known specialists than in large university hospitals with ever-changing team members.

The interaction with other MDT members enhanced the quality of the relationship itself. Personally knowing your interlocutor, as is the case in the smaller hospitals, helps to overcome some difficulties (e.g. interprofessional hierarchy) between GPs and specialists. The interaction also influenced a GP’s perception of his tasks and role and, therefore, enhanced his readiness towards future participation.

The opportunity and affordance of playing this role, however, depends on task agreements between the GP and the specialists during the meeting. These agreements are to be negotiated for every situation and patient individually. A major task agreement is on communicating MDT meeting decisions to the patient—what has to be said and by whom—to avoid conflicting or mixed messages.

The benefit of direct discussion and reaching a consensus during the meeting compared with the one-way communication of referral letters equally promotes GPs’ participation.

(3) *GPs’ role as communicators and executors of meeting decisions concerning the patient.* GPs consider the communication of meeting decisions an important responsibility, in terms of providing answers to the patient’s questions by bringing back new information; enhancing treatment adherence by advocating the chosen treatment options; and ensuring the patient’s understanding of the disease and treatment by providing adequate explanation. This conversation is to be seen in terms of continuity with previous conversations with the patient before the meeting. Most GPs preferred to take up this role, positioning this in their relationship with the patient and safeguarding the communication continuity they have with them. Others accepted this as a specialist role in cases where the patient was hospitalized during the time of the MDT meeting.

An extra motivation to attend the MDT meeting was the GPs’ need for timely and detailed information on the MDT conclusions to ensure adequate and comprehensive organization of home care for patients who were discharged from hospital, including technical aspects of treatments (e.g. stoma care), practical aspects to respond to patient disabilities (e.g. home care nurses) and treatment side effects which can be prevented or better managed if known about beforehand. In their opinion, the written meeting report does not sufficiently provide the necessary information required to organize good care.

(4) *Organizational issues for the participation of the GP in MDT meeting*. Participants differed in their preferences as to how the invitation should be delivered to the GP (by email or by telephone), but there was a consensus that it needed to be timely. For most GPs, this was for the practice to be organized; for others, it would offer the opportunity to prepare for the meeting and even to talk with the patient beforehand and clarify the patient’s view.

During the meeting, the patient’s case is in general presented by the specialist in charge of the patient. Thereafter others, including the GP, bring in additional information to deliberate on treatment plans. As time management is very important for GPs, efficient organization of the MDT meeting is fundamental to them: making sure that the necessary specialists are present, giving priority to patients of participating GPs and structuring the discussion.

MDT meetings through video conferencing were positively evaluated. Two participants had experience of it, although the majority of MDT meetings are currently co-located. The most positive attribute of video meeting is its limited impact on practice organization and time loss. Those participants who had experience of using video meetings expressed a preference towards expanding their use. However, other participants who had no experience of video meetings were less in favour, fearing the loss of direct interprofessional contact in this way. The direct personal contact had previously been mentioned as a strong benefit of co-located MDT meetings. To the GPs, it is not clear if this loss is being outweighed by the practical benefits of video MDT meetings.

## Discussion

### Main findings

The main findings are:

1. Attending an MDT meeting is perceived as part of the GP’s work, especially for complex situations.
2. Continuity of care should be the focus of collaboration.
3. Interprofessional collaborative relationships and the GP’s perceived benefit to the MDT meeting are important motivators to participate.

### Strengths and limitations

This is the first time that GPs’ preferences and experiences regarding MDT meetings in oncology have been described in the Belgian healthcare system. We cannot exclude social desirability in some answers, as the peers interviewed the participants. Our study is based on the views of GPs on the MDT meeting experience although as a recent survey has shown [6], only a minority of GPs is attending (about four per cent of all MDT meetings). Therefore, our results are not generalizable to the whole group of GPs. A separate interview study investigating the views of GPs without MDT meeting experience might complement this study.

### Interpretation of the results in relation to existing literature

Although this study has been conducted in one country, the results point to several non-contextual variables affecting the process and outcome of MDT meetings. As such, this study can be of interest internationally, as becomes clear through the comparison with existing literature calling for clarity on GPs’ role in cancer care [12–14].

### MDT meeting attendance is part of the GP’s job

Participants regarded attending MDT meetings as part of their work, though the degree of complexity needs to be high enough to be motivated to attend the MDT meeting. Case complexity can present itself in several ways: high technicity or complex chemotherapy regimens, or complex psychosocial factors and home care organization [15]. Each case has to be evaluated individually.

### Continuity of care as part of the GP’s mission

Literature stresses that physicians accept responsibility for continuity of care [16]. Participants in our study also mentioned continuity as an argument for attending MDT meetings. This relates to a definition of continuity of care given by Haggerty and colleagues: ‘Continuity is the degree to which a series of discrete healthcare events is experienced as coherent and consistent with the patient's medical needs and personal context’ [17].

Care continuity is regarded as being one of the GP’s main foci, as described by the World Organization of Family Doctors (Wonca). Wonca suggests six competence areas as being fundamental in the delivery of high-quality primary care: (1) holistic modelling; (2) person-centred care; (3) primary care management; (4) a comprehensive approach; (5) specific problem-solving skills; and (6) community orientation. All of these were mentioned by the participants in our study as contributing in their motivation to attend the MDT meetings. Therefore, we can state that attending MDT meetings harmonizes with the GPs’ general mission in healthcare.

### Information flow during the care continuum

A major topic of concern within the care continuum is the information continuum. The patient’s history must inform the MDT meeting discussion, which in turn must shape the organization of future care. The optimization of this information flow involves multiple levels: preparedness to share information, organizational aspects of the meeting providing time and space, and the practical requirements for managing all the information in an accessible way, e.g. a shared online Electronic Health Record [18]. Embedded in the GP-patient relationship, some state a preference for communicating the MDT meeting results to the patient instead of having them delivered by the specialist. Patients want their GP to be more involved in their cancer care, although both share a concern towards acquiring and maintaining the necessary skills, including communication skills [19].

Other studies confirm the task of a GP during cancer follow-up as being a flexible mediator (between patient and clinic, interpreting and translating), an efficient handyman (solving practical problems) and a personal companion throughout the illness [20].

This positions the GP as a guardian of the information on patients’ needs and context at the centre of the MDT dynamics.

### Case complexity

Our study shows that the GP’s role of information manager is especially relevant in complex cases. In less complex cases, a distinct way of sharing information could be preferred [8]. Healthcare professionals such as specialized nurses in oncology or MDT meeting organizers could call the GP before the meeting with specific questions. These questions may relate to the patient’s preferences or to specific aspects of home care, so that the GP can fulfil his/her role without being present. This is a timesaving procedure for all physicians involved. However, the benefit of a group discussion with other disciplines is lost in this way [21]. There are, however, differing views towards the benefit of meeting each other in person, ranging from preferring to participate in person, through video meeting, to preference for a private telephone call to the specialist. Exploring possible strategies for selecting appropriate patient cases for in-person MDT meetings might benefit the tight schedule and speedy meetings.

Positive interactions during the meetings are based on good interprofessional relationships, enhancing GPs’ efforts to attend. It might be helpful—e.g. by organizing joint CME sessions—to foster interprofessional relationships. Literature shows that interprofessional education leads to better interprofessional collaboration, which in turn leads to better patient care [22,23].

### Barriers for attending MDT meetings

Even though our participants regarded attending MDT meetings as part of their work, in reality the number of attending GPs is very low and in strong contrast with this view. The described barriers are mostly practical or logistical in nature, which can be overcome by video conferencing [13]. Our study suggests that a timely invitation, offering the GP enough time and opportunity to contact the patient and discuss his/her will and treatment preferences before the MDT meeting, might reinforce GPs’ participation and might benefit the quality of GPs’ input. However, some cases require urgent meeting scheduling, hindering timely invitation resulting in GPs' being absent or attending the meeting in an unprepared way. Addressing these practical issues shows the organizers’ knowledge and respect for the GPs’ situation and leads to more efficient collaboration [24]. None of the GP variables used during the sampling procedure had a clear effect on the MDT attendance. Small hospitals seem to attract GP involvement in MDT meetings more than large university hospitals, as GPs experience a lower threshold to engage in the interprofessional network from the former.

### GPs’ self-perceived benefit to the MDT meetings

GPs’ self-confidence about their contribution to the MDT meeting process varies. MDT meetings are often perceived as being focused on medical expertise. Some participants found it difficult to bring non-medical information into the discussion, thereby confirming the literature [25]. This might be even more difficult if the GP is not sure whether he/she represents the patient’s view, due to lack of a written statement from the patient (e.g. Advance Care Planning documents).

In the complex situation of cancer care, the collaboration between GPs and specialists is a necessity. GPs spontaneously respond to this need by directly contacting the oncologist and using networks of trusted providers [14]. In our study, those informal contacts remain important and are complementary to the MDT. Building those professional networks is even stated as an explicit outcome of the meetings.

#### Implications for research and/or practice

The synergy and complementarity between GPs and specialists have to be highlighted. The MDT meeting’s organizer, specialists and GPs need to be informed and convinced of the benefit of sharing expertise. The meeting process would benefit from a clarification of each participant’s role [14,15].

Some important patient information (functional status, patient’s wishes) necessary for the organization of home care could be systematically integrated into the MDT meeting discussion and into the meeting report. GPs suggest a more detailed report, including specific information relating to their role: information for the patient (what has to be said and by whom) and information for the organization of care at home (what is needed and what can be expected). Other studies have emphasized the importance of good communication and claim that the report needs a good structure [21,26,27]. In the MDT meeting process, as in general, there is an opportunity for GPs and specialists to reach an agreement on what a report should include [28]. Standardizing the MDT meeting report with the aid of a shared electronic patient health record might optimize the efficiency of the information flow.

Some advantages have been mentioned about video meeting: more GPs would prefer video meetings due to the lack of interference with practice organization. A prerequisite for the effective implementation of a video meeting is the use of a uniform and simple software package.

An MDT meeting is an operationalization of a good concept: interprofessional patient care. Embedding the MDT meeting in the interprofessional oncological care continuum might raise the awareness and commitment of all participants to enhance the effectiveness of the entire care process. This striving towards high quality seamless palliative care fits into the currently developing models of integrated care. This is a focus of models of integrated care and should be further evaluated [29].

## Conclusions

General practitioners perceive attendance of MDT meetings as an integral part of their job. Case complexity and good interprofessional relationships are strong facilitators for GPs’ attendance. Ensuring a smooth information flow and addressing practical barriers for attendance—e.g. by installing video conferences—were mentioned as areas for improvement.

## Appendix A. Interview guide.

**Table:**

Topic	Content	Question
The MDT meeting in theory	What does the concept ‘MDT meeting’ mean?	If I say ‘MDT meeting,’ what do you think of? How would you define an MDT meeting?
Experience	What is your experience of MDT meetings? How do the meetings work out? What is the continuation of the MDT meeting?	How frequently do you participate in MDT meetings? Do you have in your region experiences with MDT meetings in multiple hospitals? If we look back at the last MDT meeting you attended, can you tell me how you experienced this? (How were you invited? How much time before the meeting? Which type of patient, type of tumour? What was the reason you attended? Is this experience representative of the experiences you have had with MDT meetings in general?) How do you attend the MDT meeting? (Being present, attending through teleconference or video conferencing?) If you attend MDT meetings in multiple hospitals, what are the main differences? Do your patients know in advance that an MDT meeting is organized? If so, how and by whom are they informed (If not, why not?) How do you experience the process of decision making during the MDT meeting? Is deliberation happening? With whom? Who is mainly speaking? Who is taking the final decision? What is your input during the MDT meeting? Which information do you receive after the MDT meeting? If you are not able to attend, how is the exchange of information about the patient done? Do you get informed about the decisions taken? How? How do you experience this? Does the patient get informed? How? How do you experience this? How is your attendance at the MDT meetings remunerated?
Perceptions	GP’s role during the MDT meeting. Positive contribution of the MDT meeting towards the patient. Positive contribution of the MDT meeting towards the GP.	What do you think other participants’ expectations of your attendance at the MDT meetings are? What is your contribution during the MDT meeting in general? Can the MDT meeting positively contribute to the course of the disease or treatment of the patient? Do you consider the MDT meeting as useful for every patient? Why (not)? Does attending the MDT meeting contribute something towards you? What?
Facilitators and barriers	GPs’ experiences of facilitators and barriers towards attending the MDT meetings (practical and organizational factors, factors concerning content and relationship).	What is your most important argument for attending the MDT meeting (or not)? What is facilitating or interfering with your attendance of the MDT meeting?
Suggestions for improvement	How could GPs’ participation at the MDT meeting be improved?	What can be a way to improve GPs’ attendance of the MDT meeting?
